# Fetal hyaloid artery in first trimester of pregnancy: slow*flow*HD study

**DOI:** 10.1515/crpm-2024-0025

**Published:** 2024-10-09

**Authors:** Toshiyuki Hata, Aya Koyanagi, Riko Takayoshi, Yasunari Miyagi, Takahito Miyake

**Affiliations:** Department of Obstetrics and Gynecology, Miyake Clinic, Okayama, Japan; Department of Perinatology and Gynecology, Kagawa University Graduate School of Medicine, Miki, Kagawa, Japan; Department of Gynecology, Miyake Ofuku Clinic, Okayama, Japan

**Keywords:** Slow*flow*HD, fetal hyaloid artery, first trimester, down syndrome

## Abstract

**Objectives:**

To detect fetal hyaloid artery (FHA) blood flow using Slow*flow*HD in the first trimester.

**Methods:**

During the 8-month period from February to September 2023, one-hundred and eighty-three trans-abdominal scans were performed for first-trimester screening in singleton pregnancies at 11–13 + 6 weeks of gestation. One-hundred and fifty-two cases were excluded from the study due to inappropriate fetal positions, excessive fetal movements, and excessive distances between the fetus and probe; thus, thirty-one fetuses (20.4 %) were examined, comprising 19 uni- and 12 bilateral orbits. One fetus with trisomy 21 was also evaluated at 12 weeks and 1 day. FHA was classified into two types based on the starting point angle (Straight, straightly diverged from ophthalmic artery; and Curved, crookedly diverged from ophthalmic artery) using Slow*flow*HD.

**Results:**

In 19 uni- and 12 bilateral orbits in 31 fetuses, FHA could be identified in all orbits. In one orbit at 13 weeks and 5 days, blood flow on the string FHA could be detected. The vasa hyaloidea propria was noted in 3 orbits, and the posterior vascular capsule was depicted in 7 orbits. The incidence of Straight-type FHA increased with advancing gestation (p<0.0001). In a fetus with Trisomy 21, Straight-type FHA was noted in the left orbit, whereas FHA could not be identified in the right orbit.

**Conclusions:**

This is the first report on the detection of FHA in the first trimester of pregnancy. Slow*flow*HD may be a useful diagnostic modality to evaluate human vascular development, such as FHA *in utero*.

## Introduction

The fetal hyaloid artery (FHA) was reportedly detectable in 90 % of fetuses by the 14th week of gestation using transvaginal two-dimensional sonography [1]. FHA was detected in all cases at 14–18 weeks of gestation, and blood flow within FHA could be noted using conventional power Doppler until the 16th week of gestation [2]. Blood flow in FHA was evident in 83.5 % using Slow*flow*HD at 18–21 + 6 weeks of gestation [3]. However, there has been no study to assess blood flow in FHA in the first trimester of pregnancy.

Slow*flow*HD can detect fetal peripheral vessels with low-velocity blood flow [4, 5]. In this study, we attempted to detect FHA blood flow using Slow*flow*HD in the first trimester of pregnancy.

## Materials and methods

During the 8-month period from February to September 2023, one-hundred and eighty-three trans-abdominal scans were performed for first-trimester screening in singleton pregnancies at 11–13+6 weeks of gestation. Crown-rump length (CRL) measurement at 8–10 + 6 weeks of gestation was conducted to confirm the fetal age [6]. Slow*flow*HD scans for FHA evaluations were conducted with Voluson E10 BT20 (GE Healthcare, Zipf, Austria) with a transabdominal transducer (GE C2-9-D, 3–9 MHz) or Voluson Expert22 BT22 (GE Healthcare, Zipf, Austria) with a transabdominal transducer (GE RM7C, 2–8 MHz). One-hundred and fifty-two cases were excluded from the study due to inappropriate fetal positions, excessive fetal movements, and excessive distances between the fetus and probe; thus, thirty-one healthy fetuses (20.4 %) were examined, comprising 19 uni- and 12 bilateral orbits. One fetus with trisomy 21 was also evaluated at 12 weeks and 1 day. The study was conducted following approval by the Ethics Committee of Miyake Clinic, Okayama, Japan. All participants provided written informed consent after a full explanation of the aims of the study.

All Slow*flow*HD examinations were carried out by one experienced sonographer (A.K.). The pulse repetition frequency (PRF) was set at 0.39–0.77 kHz and the wall motion filter to ‘low1’. The Thermal Index (TI) was set at 0.5 to 1.0, and Mechanical Index (MI) ranged from 0.6 to 1.0. The total examination time was within 2 min. The number of data acquisitions was 1–10.

**Figure 1:**
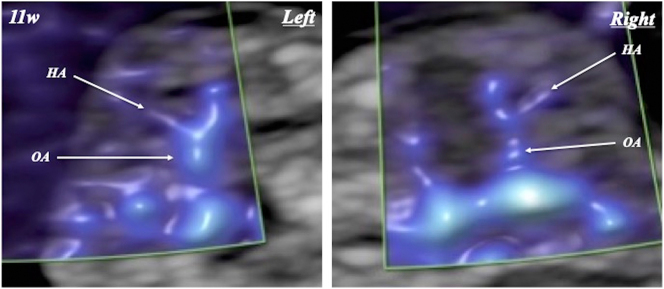
Fetal hyaloid artery (HA) detected by slow*flow*HD at 11 weeks of gestation. OA, ophthalmic artery.

**Figure 2:**
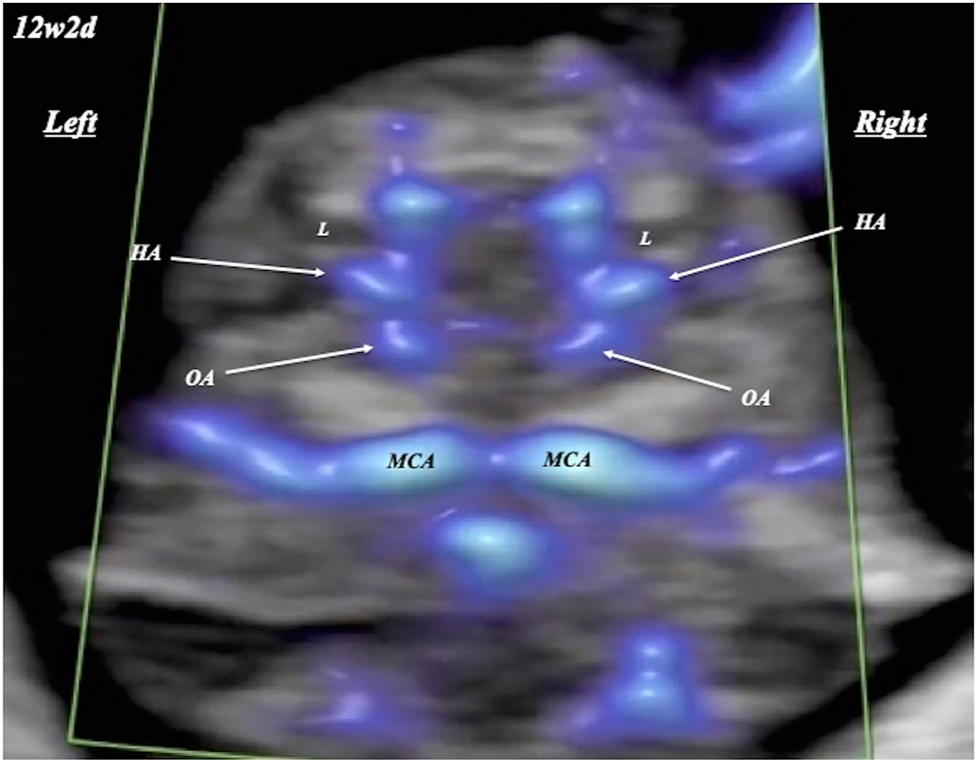
Fetal hyaloid artery (HA) detected by slow*flow*HD at 12 weeks and 2 days of gestation. L, lens; MCA, middle cerebral artery; OA, ophthalmic artery.

**Figure 3:**
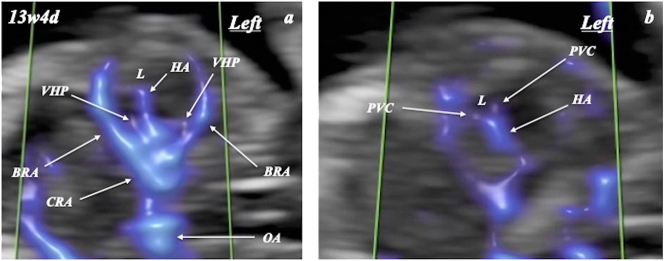
Fetal hyaloid artery (HA) detected by slow*flow*HD at 13 weeks and 4 days of gestation. BRA, branch retinal artery; CRA, central retinal artery; L, lens; MCA, middle cerebral artery; OA, ophthalmic artery; PVC, posterior vascular capsule; VHP, vasa hyaloidea propria.

**Figure 4:**
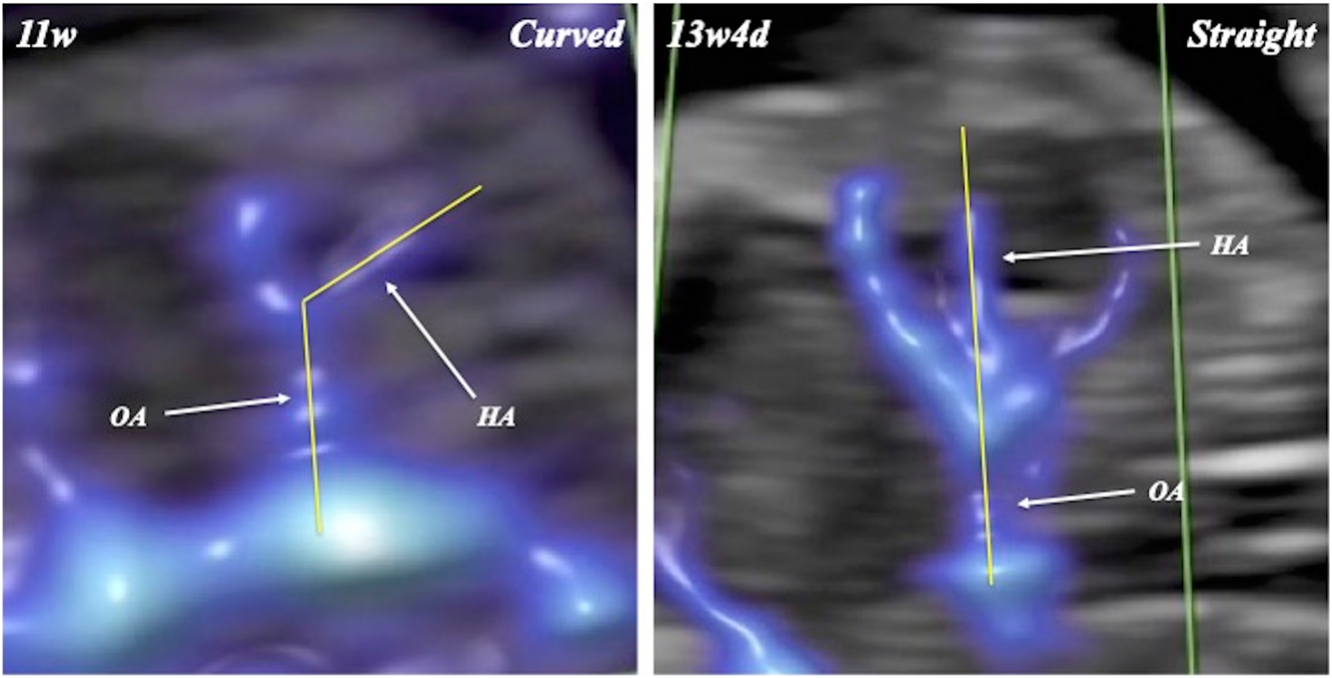
Starting point angle of fetal hyaloid artery (HA). OA, ophthalmic artery.

FHA was visualized and evaluated by two examiners (T.H. and A.K.), with complete agreement between them (Figures 1, 2, and 3). FHA was classified into two types based on the starting point angle (Straight, straightly diverged from ophthalmic artery; and Curved, crookedly diverged from ophthalmic artery) using Slow*flow*HD (Figure 4). Cohen’s kappa coefficients for FHA-type classification for intra-observer (A.K.) and inter-observer agreements were 0.903 (almost perfect agreement) and 0.763 (substantial agreement), respectively [7].

FHA types among gestational ages were analyzed with the Cochran Armitage Linear Trend test, and each FHA type among gestational ages was analyzed with ANOVA. All analyses were performed with Mathematica 13.0.0.0 (Wolfram Research, Champaign, IL, USA). A p-value <0.05 was considered significant.

## Results

**Figure 5:**
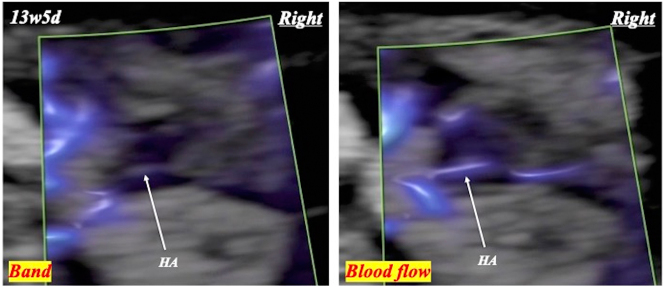
Blood flow of the string fetal hyaloid artery (HA) detected in one orbit at 13 weeks and 5 days.

**Figure 6:**
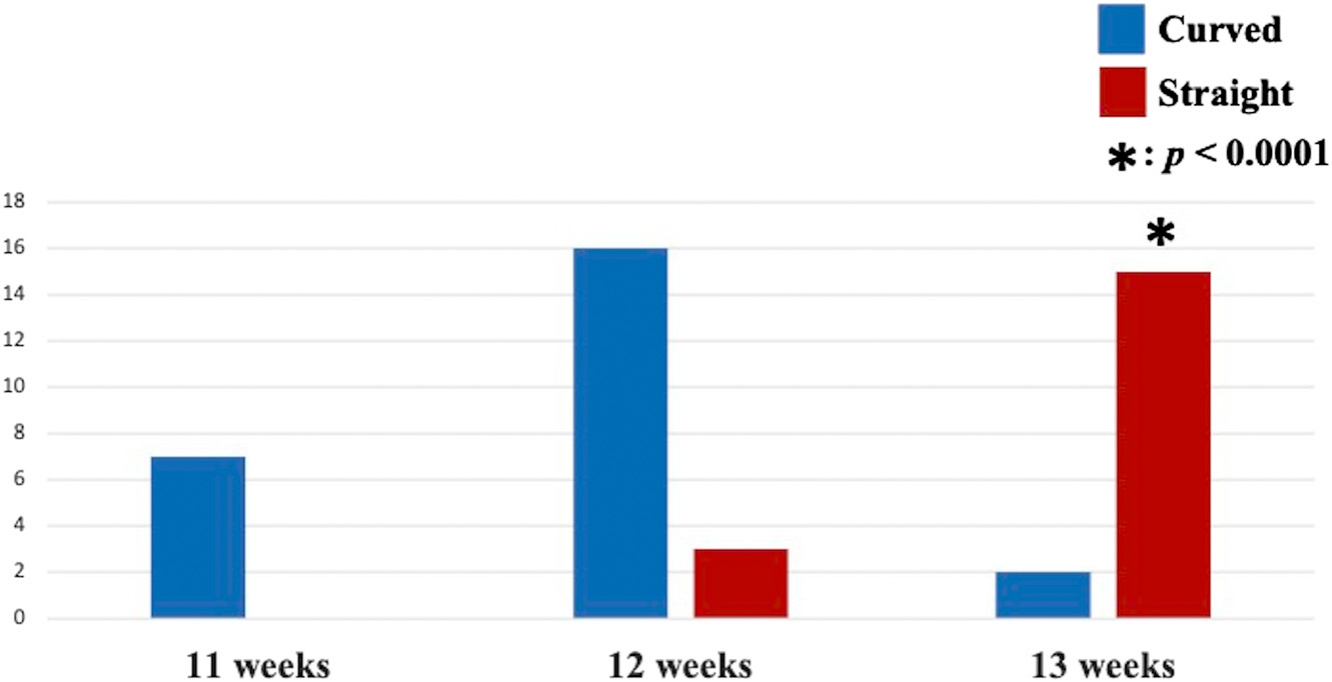
The incidence of curved- and straight-type fetal hyaloid arteries at 11–13 + 6 weeks of gestation.

In 19 uni- and 12 bilateral orbits in 31 fetuses, FHA could be identified in all orbits (Figures 1, 2, and 3 and Table 1). In one orbit at 13 weeks and 5 days, blood flow of the string FHA could be detected (Figure 5). The vasa hyaloidea propria was noted in 3 orbits, and the posterior vascular capsule was depicted in 7 orbits (Figure 3 and Table 1). The incidence of Straight-type FHA increased with advancing gestation (Figure 6 and Table 2) (p<0.0001).

**Table 1:** Detectioon of fetal hyaloid artery at 11–13 + 6 weeks of gestation.

Orbit	n	FHA				
		Band	Blood flow		VHP	PVC
			Left	Right		
Bilateral	12	0	12	12	0	1
Unilateral	19	1	10	9	3	7

FHA, fetal hyaloid artery; VHP, vasa hyaloidea propria; PVC, posterior vascular capsule.

**Table 2:** Starting point angle of fetal hyaloid artery at 11–13 + 6 weeks of gestation.

GA, weeks	n	Starting point angle of hyaloid artery	
		Curved	Straight^a^
11	7	7	0
12	19	16	3
13	17	2	15

^a^p<0.0001. GA, gestational age.

**Figure 7:**
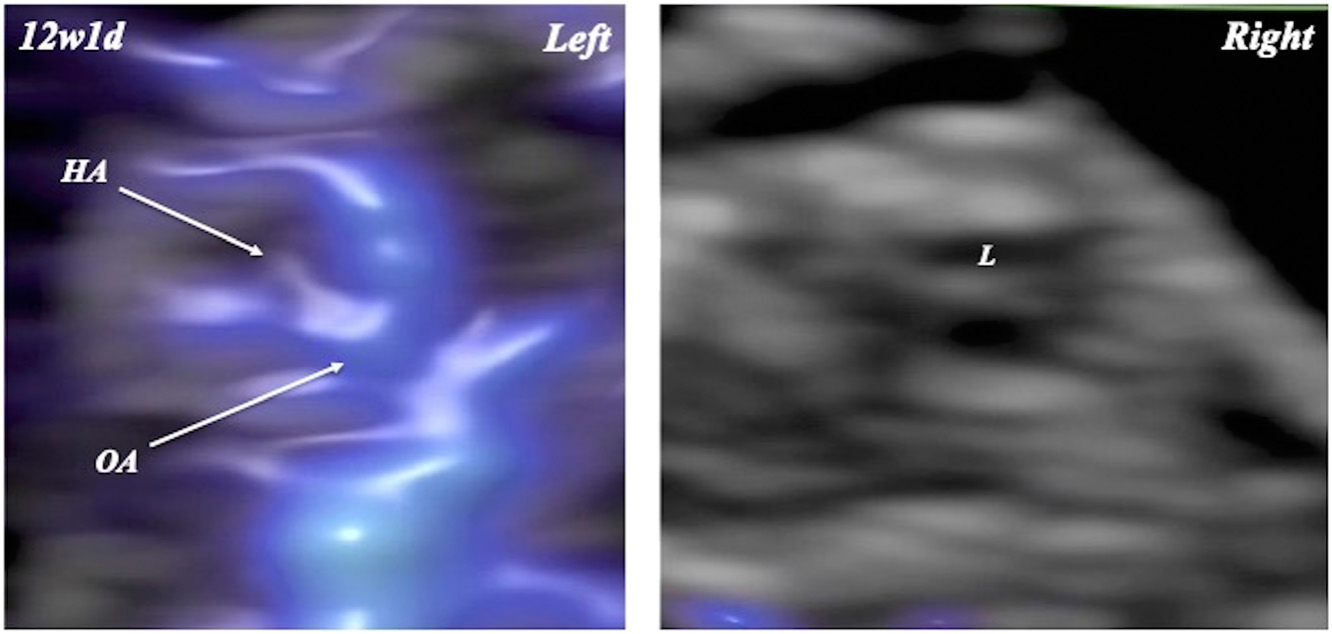
Fetal hyaloid artery (HA) in a fetus with trisomy 21 at 12 weeks and 1 day of gestation. Straight-type fetal HA can be noted in the left orbit, whereas HA cannot be identified in the right orbit. L, lens; OA, ophthalmic artery.

In a fetus with Trisomy 21 at 12 weeks and 1 day of gestation, straight-type FHA was noted in the left orbit, whereas FHA could not be identified in the right orbit (Figure 7).

## Discussion

Using conventional power Doppler, FHA blood flow could be identified between 14 and 16 weeks of gestation [2]. However, to our knowledge, the current study is the first report on the detection of FHA in the first trimester of pregnancy, and FHA blood flow could be depicted using Slow*flow*HD in all 43 orbits studied at 11–13 + 6 weeks of gestation. Moreover, the vasa hyaloidea propria was identified in 3 out of 43 orbits (7 %). The role of the vasa hyaloidea propria is blood supply to the primary vitreous [8]. In our previous investigation using Slow*flow*HD, the vasa hyaloidea propria was noted in only 4.9 % at 18–21 + 6 weeks of gestation, whereas these vessels could not be recognized at 28–31 + 6 weeks [3]. In this study, blood flow on the band of FHA could be detected. The change from the blood flow type of FHA to the band type was noted in 13.6 % of cases studied with advancing gestation [3]. This case suggests the simultaneous transition from the blood flow type of FHA to band type in the same vessel in the first trimester. Moreover, in this study, we identified two types of FHA: Curved- and Straight-types. The incidence of Curved-type FHA decreased, and Straight-type FHA increased during this short first-trimester period. The lens diameter/orbital diameter decreased with advancing gestation [9]. Morphological change in the fetal orbit during pregnancy may explain the change in FHA running. Slow*flow*HD should provide unique and novel information on human eye development in the first trimester of pregnancy.

Birnholz and Farrell [10] suggested that delayed regression of FHA occurs in trisomy 21. However, the number of trisomy 21 cases was very small in their study. In the present study, FHA could not be detected in the unilateral orbit in the case with trisomy 21 in the first trimester of pregnancy. Many children with Down syndrome had hyperopia (69.1 %) and astigmatism (58.5 %) [11]. Our findings may be related to ocular disorders in children with trisomy 21. However, the data and their interpretation should be taken with some degree of caution because of the small number of Down syndrome cases studied. Further studies involving larger sample sizes of Down syndrome cases are mandatory to confirm FHA regression during pregnancy in fetuses with trisomy 21.

With respect to the safety of the use of Slow*flow*HD during pregnancy, TI values of the Slow*flow*HD modality were within recommended values set by the World Federation for Ultrasound in Medicine and Biology, American Institute of Ultrasound in Medicine, and British Medical Ultrasound Society [12]. In the present study, TI and MI values during Slow*flow*HD examinations were≤1. The SlowflowHD examination time (2 min) in our study was very short, and scanning was performed in accordance with the ALARA principle (examining with exposure As Low As Reasonably Achievable) 13], [14], [15. Moreover, a fetal eye may be more effectively cooled due to the surrounding amniotic fluid environment [16].
